# Conservation on International Boundaries: The Impact of Security Barriers on Selected Terrestrial Mammals in Four Protected Areas in Arizona, USA

**DOI:** 10.1371/journal.pone.0093679

**Published:** 2014-04-09

**Authors:** Jamie W. McCallum, J. Marcus Rowcliffe, Innes C. Cuthill

**Affiliations:** 1 School of Biological Sciences, University of Bristol, Bristol, United Kingdom; 2 Institute of Zoology, Zoological Society of London, Regents Park, London, United Kingdom; 3 Conservation Programmes, Zoological Society of London, Regents Park, London, United Kingdom; University of Marburg, Germany

## Abstract

Several thousand terrestrial protected areas (PAs) lie on international boundaries. Because international boundaries can be focal points for trade, illegal activity and development, such PAs can be vulnerable to a range of anthropogenic threats. There is an increasing trend towards the erection of international boundary infrastructure (including fences, barriers and ditches) in many parts of the world, which may reduce the risk of these anthropogenic threats to some PAs. However this may restrict home range and access to resources for some native species. We sought to understand the impacts of these two different types of threat by using camera traps to measure the activity level of humans, native and invasive mammals in four US PAs on the Mexican international boundary. Comparisons were made between treatment areas with barriers and those without. Results showed that puma and coati were more likely to appear in treatment areas without barriers, whereas humans were not observed more frequently in one treatment area over another. The suggestion is that the intermittent fencing present in this part of the world does affect some native species, but does not necessarily restrict the movement of humans (including illegal migrants), who may negatively impact native species.

## Introduction

Protected areas (PAs) can help safeguard biodiversity from several anthropogenic threats [1], [2], [3], [4], [5], [6]. The UNEP World Conservation Monitoring Centre (UNEP-WCMC) has identified 3043 PAs, making up 227 international boundary clusters, which are part of existing or potential transboundary conservation units [7]. There are many more PAs adjoining international boundaries (PAAIB) that are not part of such units, for example the Buenos Aires National Wildlife Refuge (BANWR) on the US-Mexico international boundary. PAAIBs are therefore a large and important part of the global PA network.

However, in a period of increasing globalisation, international boundaries and frontier zones are becoming more highly populated areas of cultural and commercial transition, regulation and development [8]. This increase in human population, development and trade can result in impacts on biodiversity from either side of the international boundary, both inside and outside PAs. The effects of these impacts may be hard to control because the source may originate in another country with different socio-economic pressures, environmental laws and enforcement capabilities. Illegal transboundary activity may also have security or political implications [8]. As a result, selecting optimal mitigation strategies for PAAIBs is an important task.

“Nature Protectionists” argue for strong maintenance of all PAs, with minimal human incursion - a version of “fortress conservation” [9]. Recent studies on African lion (*Panthera leo*) across 11 countries, show the benefit of fenced-in populations for large carnivores [10]. In the PAAIB context, escalated defensive measures (driven by biodiversity and/or security preoccupations) may result in a “thickening” of the boundary [11]. Militarised, policed and even economic boundaries are generally marked with linear barriers or buffers. These are often accompanied by roads, ditches, marker posts or vegetation clearance. In combination with these tangible expressions of the boundary, different states may manage it with differing degrees of legal, political and law enforcement intensity to meet their objectives. These will dictate the ease with which international boundaries can be crossed by wildlife, goods and people [12]. These measures may restrict transboundary human and invasive access to the PAAIBs, thereby reducing impacts on biodiversity. However such “thickening” may also exacerbate habitat loss, degradation and subdivision [13]. In doing so, it may disrupt natural processes, flows and species distributions, colonisation and pollination, possibly leading to a cascade of negative effects [8]. Furthermore linear infrastructure may only displace impacts on biodiversity to neighbouring spaces [3], leading to isolation of the PA [1], [2], [14]. “Thickening” strategies may even increase opportunities for human access (through service roads and construction processes), with the potential to stimulate long-term invasive species activity or increased pollution. In response to Packer et al. [10], Creel et al. [15] list and analyse these threats in greater detail.

However it remains practically impossible to measure biodiversity, threats and solutions in a PAAIB both with and without a “thickened” boundary in the same place at the same time. As a result the best approach is difficult to identify. In the absence of this clear evidence, relevant management decisions are often shaped by socio-economic, political and security agendas and by individual subjectivity [16].

Because of resource limitations, it is important to identify conservation approaches that are likely to be successful [17]. This research is directed at understanding whether “thickening” infrastructure on an international boundary perimeter of a PA is likely to inhibit movement and access to resources for certain native species, as well as preventing impacts from humans and invasive species [18].

In a broader theoretical context it is hoped that the analysis will help to advance knowledge in the fenced reserves debate [10], [15], [19]. Much of this discussion is focused on whether it is better to expose native species to invasive threats while providing them with access to extensive habitat and resources, or to protect them with barriers, but isolate them, thereby reducing their dispersal ability and access to resources.

Casting light on these research questions is intended to help PAAIB managers and policy makers to direct their resources effectively, or at least enable them to present an informed ecological case when considering the impacts of international boundary infrastructure on biodiversity. This will help in the planning, funding, transboundary coordination and management of such sites for the benefit of biodiversity protection. These questions need to be addressed with some urgency because of increases in biodiversity loss and international boundary thickening [20]. Immigration legislation passed by the US Senate in June 2013 [21], focuses on increased border security between the US and Mexico and instructs the Department of Homeland Security to prepare a report on increased infrastructure, with a budget of US$ 1.5 billion allocated for its installation [21].

## Methods

### Ethical statement

The research was approved by the Ethical Review Process of the University of Bristol (Ref: UB/08/046) with an expiry 26/02/2012, by which time all field work had been conducted. The ethical approval application specified non-invasive camera trap data collection in relation to terrestrial mammals over 10 kg and the measurement of illegal human activity. Only presence-absence data was of interest and therefore analysis was carried out anonymously. Permits were granted at all four protected areas; by Fish and Wildlife Service at BANWR (Permit: 22s30 2010-008), US Forest Service at NRD (Authorization ID: SUP0109, FS-2700-25 (03/06)) and the National Park Service at CNM (Permit: CORO-2008-SCI-0002) and ORPI (Permit: ORPI-2007-SCI-0014).

Native species, human and alien invasive detection counts were taken in four PAAIBs. These were measured against the type of international boundary infrastructure to explore effects on each of the target groups.

### Study areas

Designing a true experiment of PAAIBs testing for the impacts of international boundary barriers over large spatial and time scales, with identical treatments, control replicates and pre-treatment conditions would be expensive, time-consuming and logistically difficult [22]. As a result we sought a location onto which the experimental design could be overlaid. Although it can be hard to control for every factor with this approach, important insights can still be attained [23], [24].

Location criteria included identification of areas that had (a) high wildlife biodiversity, in order to be able to measure target groups and detect any variations, (b) likely transboundary activity from the three target groups, (c) differing degrees of international boundary infrastructure to enable comparisons and (d) sufficient number of PAAIB replicates to generate adequate power for statistical analysis.

The state of Arizona shares a 626 km international boundary with the state of Sonora in Mexico. Both the US and Mexico rank in the top ten nations for biological diversity and are included in the group of megadiverse nations by Conservation International [25]. On a more localised scale two of the top ten most biodiverse counties (Pima and Cochise) in the continental US sit on the Arizona-Sonora international boundary [8] including a high number of biodiversity islands [8]. These include the geological formations known as the “Sky Islands” or Madrean Archipelago - a chain of 42 forested mountain peaks that rise out of cactus scrub plains; 27 of them in the US and a further 15 in Mexico. Four PAAIB sites within this area possess each of the criteria outlined above.

**Figure 1:**
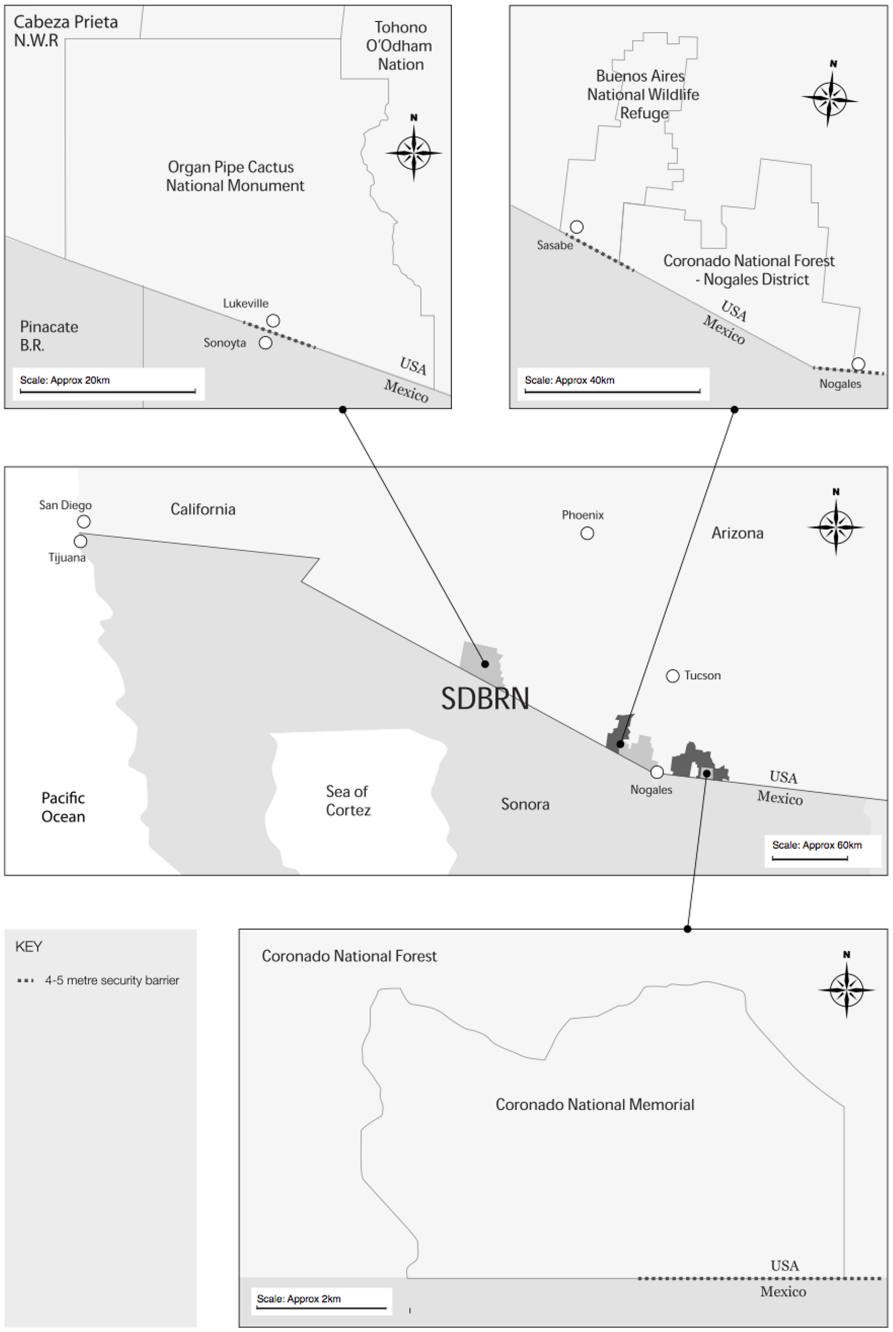
Sonoran Desert map. Four PAs surveyed, including position of linear boundary infrastructure.

Organ Pipe Cactus National Monument (ORPI) lies in Pima County, Arizona and is a 1322.06 km^2^, IUCN Category III Natural Monument and UN International Biosphere Reserve, run by the National Park Service (NPS). Its ecoregion category is “Sonoran Basin and Range” [26] and “American Semi-Desert” [27]. Buenos Aires National Wildlife Refuge (BANWR) lies in Pima County, Arizona and is a 473.9 km^2^, IUCN Category IV Habitat Species Management Area managed by the US Fish and Wildlife Service (USFWS). Its ecoregion category is “Madrean Archipelago” [26] and “American Semi-Desert” [27]. The Nogales Ranger District of the Coronado National Forest (NRD) is an IUCN Category V Protected Landscape in Santa Cruz county, Arizona. It is one of five sections of the Coronado National Forest. Its main component is the Tumacacori National Forest Reserve which is 823.7 km^2^ and is managed by the US Department of Agriculture, Forest Service (USFS). Its ecoregion category is “Madrean Archipelago” [26] and “American Semi-Desert” [27]. Coronado National Memorial (CNM) is an IUCN Category iii National Monument or Feature, situated in Cochise County, Arizona. It is managed by the National Park Service and covers 20 km^2^. Its ecoregion category is “Madrean Archipelago” [26] and “Chihuahuan Scrub” [27]. All four sites have overlapping habitat types and three lie within the Madrean Archipelago. Each adjoin an international boundary, with at least 1 km of 4–5 m non-porous steel barriers. Transboundary human activity and extensive law enforcement counter-measures are present at each site (Figure 1).

The four PAs have similar habitat on the other side of the international boundary, although three of them lie within 10 km of towns in Mexico. In each case there is no PA south of the boundary, although the buffer area of ORPI does touch on the buffer area of El Pinacate Biosphere Reserve. However these contiguous boundaries are divided by the large east-west highway Route 2 in Mexico.

### Data collection

Although habitat research can benefit from the study of several taxa [28], [29], it is not feasible to collect data on every species. As a result data were collected on species likely to have a wider impact on the entire ecosystem. These included those that might interact with others through competition [30], predation [30], mutualism [31], disease [32], facilitation [33], enrichment and ecosystem engineering [34].

Because barriers that dissect, filter, eliminate or complicate movement [35] can influence large and small mammals [36] as well as carnivores [37], we decided to focus on native Artiodactyla and Carnivora (Table 1).

**Table 1:** Target species for camera trap investigation in four Arizona protected areas adjoining international boundaries, 2010–2011.

Species common name	Latin name	Order	Family
**Native Species**			
American black bear	*Ursus americanus*	Carnivora	Ursidae
Bobcat	*Lynx rufus*	Carnivora	Felidae
Puma	*Puma concolor*	Carnivora	Felidae
Coyote	*Canis latrans*	Carnivora	Canidae
Gray fox	*Urocyon cinereoargenteus*	Carnivora	Canidae
Kit fox	*Vulpes macrotis*	Carnivora	Canidae
Coati	*Nasua narica*	Carnivora	Procyonidae
Raccoon	*Procyon lotor*	Carnivora	Procyonidae
Ringtail	*Bassariscus astutus*	Carnivora	Procyonidae
American Badger	*Taxidae taxus*	Carnivora	Mustelidae
Western hooded Skunk	*Spilogale gracilis*	Carnivora	Mephitidae
Hooded skunk	*Mephitis macroura*	Carnivora	Mephitidae
Striped skunk	*Mephitis mephitis*	Carnivora	Mephitidae
Common hog-nosed skunk	*Conepatus mesoleucus*	Carnivora	Mephitidae
White tailed deer	*Odocoileus virginianus*	Artiodactyla	Cervidae
Mule deer	*Odocoileus hemionus*	Artiodactyla	Cervidae
Collared Peccary	*Pecari tajacu*	Artiodactyla	Suina
**Invasive species**			
Cattle	*Bos taurus*	Artiodactyla	Suina
Domestic dog	*Canis familiaris*	Carnivora	Canidae
Horse	*Equus caballus*	Perissodactyla	Equidae

The level of transboundary biodiversity impacts in PAAIB were assessed by proxy by counting the number of human detections through the use of camera traps. While human detection cannot account for the absolute levels of biodiversity impacts when counted inside the PA, it can give an indication, especially in a spatially comparative study.

Humans were also categorised according to the following identification protocols.

1. If they wore an official US government agency uniform (CBP, NPS Ranger, Army) they were classified as “Law Enforcement” (LE).
2. If they were ***not*** classified LE and carried hessian-bale backpacks they were classified as “Smugglers”
3. If they were ***not*** LE and ***not*** smuggler and carried makeshift water bottles (soft-drink containers) and backpacks (urban daypacks), and wore non-specialised hiker's clothing and footwear, they were classified as undocumented alien (UDA).
4. All other human detections were classified as miscellaneous. These included possible ranchers, hunters and hikers and those that were hard to classify.

We classified LE in order to ensure that they were not counted towards other groups. However, we did not analyse them against the treatments or other species in a bid to keep their activity patterns confidential. We did however include their numbers in the counts and analyses of total human activity.

In addition to the proxy measure of human activity for biodiversity impacts, it was desirable to measure invasive species activity directly. It was not possible to count all invasive species, so a representative group was chosen which could be counted using the same methods as the native species counts. Therefore invasive Artiodactyla and Carnivora (target invasive species - Table 1) were chosen as representative Orders. In the region studied, this included livestock such as cattle and horses as well as feral species such as dogs.

The activity of these three target groups was measured through detection or non-detection counts using camera traps. These counts were used as the main measure for treatment analysis. For some tests (e.g. co-occupancy) these counts were also used to measure and compare target groups in different areas over different time-spans by dividing the total number of detections by the total number of survey periods. In this study and effort included a 24 hour period and the resulting figure was multiplied by 100 to give a trap rate per 100 days (pcd).

Within each of the four PAAIB, detection counts were collected in three separate treatment zones, representing areas that were as similar as possible with the exception of international boundary barriers: P (porous, open, unbarriered boundary areas), NP (non-porous, closed, barriered boundary) and BE (barrier-end representing the first 500 m of open territory after a barrier end). The latter category was included because, near the ends of barriers, there may be increased movement of animals and people, diverted by the barrier.

Between May 2010 and March 2011, 36 camera traps were deployed, with three in each of the three treatment zones in each of the four PAs. These cameras included 12 Reconyx Rapidfire Covert RCR 60 cameras (Reconyx Inc. Holmen, USA) and 24 Reconyx HC600 color infrared trail cameras. They were placed 1–2 km north of the international boundary in north/south running dry river beds (arroyos) which showed signs of target native species, target invasive species and humans (Table 2). By substituting space for time (comparative sites rather than before-after tests) [24] it was possible to compare target species activity in similar habitat in the same PA but in different treatment conditions.

**Table 2:** Camera trap coordinates, altitude, distance to international boundary and nearest camera in four Arizona protected areas adjoining international boundaries 2010–2011.

Camera code	Latitude	Longitude	Altitude (m)	Boundary dist. km	Next camera km
01.ORPI.NP1	31.884774	−112.787651	436	1.41	0.53
02.ORPI.NP2	31.887300	−112.782803	438	1.83	0.53
03.ORPI.NP3	31.884836	−112.777678	439	1.75	0.56
04.ORPI.BE1	31.879217	−112.768497	440	1.43	0.61
05.ORPI.BE2	31.881800	−112.763000	444	1.87	0.61
06.ORPI.BE3	31.878800	−112.757286	444	1.74	0.63
07.ORPI.P1	31.874600	−112.732900	451	2.00	0.85
08.ORPI.P2	31.868800	−112.726761	449	1.72	0.51
09.ORPI.P3	31.866662	−112.721897	450	1.59	0.51
10.BA.NP1	31.478000	−111.493047	1061	1.22	1.00
11.BA.NP2	31.476558	−111.480174	1082	1.49	0.55
12.BA.NP3	31.471588	−111.479990	1075	1.00	0.55
13.BA.BE1	31.477370	−111.467369	1093	1.98	0.87
14.BA.BE2	31.469359	−111.467450	1086	1.15	0.80
15.BA.BE3	31.471000	−111.458653	1112	1.58	0.80
16.BA.P1	31.458904	−111.429925	1142	1.29	0.59
17.BA.P2	31.461200	−111.424397	1154	1.74	0.59
18.BA.P3	31.456898	−111.421301	1141	1.37	0.60
19.NRD.P1	31.349303	−111.070328	1387	1.91	0.50
20.NRD.P2	31.344700	−111.069931	1454	1.37	0.50
21.NRD.P3	31.343315	−111.016078	1401	1.22	0.89
22.NRD.BE1	31.346704	−111.023013	1251	1.60	0.73
23.NRD.BE2	31.340173	−111.021953	1300	0.87	0.55
24.NRD.BE3	31.340598	−111.012000	1285	0.95	0.55
25.NRD.NP1	31.347846	−111.007584	1249	1.73	0.92
26.NRD.NP2	31.350072	−110.997763	1248	1.96	0.54
27.NRD.NP3	31.349618	−110.992856	1241	2.00	0.54
28.CNM.P1	31.346380	−110.302000	1698	1.45	0.50
29.CNM.P2	31.350403	−110.297839	1721	1.90	0.50
30.CNM.P3	31.346134	−110.296644	1714	1.43	0.50
31.CNM.BE1	31.346984	−110.264213	1648	1.50	0.68
32.CNM.BE2	31.344761	−110.257322	1616	1.29	0.35
33.CNM.BE3	31.344331	−110.254067	1620	1.25	0.35
34.CNM.NP1	31.347586	−110.245611	1717	1.57	0.51
35.CNM.NP2	31.344980	−110.239759	1555	1.43	0.50
36.CNM.NP3	31.346078	−110.233375	1535	1.39	0.67

*P = porous, NP = non-porous, BE = barrier-end. ORPI = Organ Pipe Cactus National Monument, BA = Buenos Aires National Wildlife Refuge, NRD = Nogales Ranger District (Coronado National Forest), CNM = Coronado National Memorial.*

To increase the likelihood of data independence, trap stations were placed a minimum of 500 m apart and the P and NP zones were over 4 km apart at their closest point. This distance takes into account the home range of most likely target species, reducing the likelihood of pseudo-replication or one detection being influenced by another. In order to observe this regime at CNM, three P zone camera traps needed to be placed just outside this PAAIB in a contiguous PAAIB (Sierra Vista Ranger District of the Coronado National Forest). This precaution was reinforced at all sites by temporal independence protocols which restricted records of a single species to one within 24 h.

The rule that camera trap data should be collected in areas frequented by target species such as dirt roads, game trails or regular travel routes [38], was observed in order to ensure a high probability of ‘capture’ if the target species was present. This is particularly important for elusive species, because without enough count data, there may not be enough power to detect differences and provide statistically valid outcomes. Camera traps were placed on the edge of arroyos with their back to the matrix in order to measure activity use within these riparian areas. These types of riparian systems (even dry ones) contain water, cover, shade, vegetation and prey [39] and were therefore considered likely to be used regularly by many of the target species. Detection counts in arroyos cannot provide an entire picture of target species activity, but can help identify a relative difference in species activity between areas, which is the focus of this research rather than an attempt to estimate population or space-abundance relationships, in which every missed detection may be critical.

During the first month of deployment, cameras were visited at least once in order to diagnose and solve any immediate problems such as poor positioning, high false triggers or exposure to disruptive environmental influences. After this time, they were visited every four to six weeks to carry out standard maintenance, battery replacement, re-position camouflage and extract images from data cards. Raw photographs were transformed into a range of ecologically relevant numerical data including detection or non-detection using published protocols [40]. Those species present at less than 1/4 of stations and which had fewer than 15 detections were disregarded as these numbers would be too low to carry out any robust statistical analysis. According to this system [40] each camera station is allocated a separate folder, which in turn contains a folder for each of the target species. Each of these folders has a separate folder for one, two, three, four and more individuals of that species. Once this system has been assembled, each photograph is renamed according to the Exchangeable Image File Format (EXIF) time and date metadata and then placed in the relevant folder. The investigator then enters DOS and locates the parent folder of these camera traps (using “chdir\foldername”). These can then be turned into a text file with the instruction: “dir/s>allfoldersfiles.txt. Inputing “allfoldersfiles.txt” then produces a text file, which lists all detections by time, date and location. This file needs to be coupled with another text file, which lists each of the target species, camera stations, their GPS co ordinates and the dates that they were operational. This is then run through a further analysis which produces an output of basic indices, including counts by day, detection times and trap rate.

### Data analysis

Counts of individual species records by treatment type were analysed using GLM, modelled as negatively binomially distributed with a log link function and the log of the number of trap days included as an offset. We used one GLM for each of the target native and invasive species as well as their family and order groups and the same for human categories (see LE exception above) and the overall human category. Models were fitted using the glm.nb function in the MASS package in R [41], [42]. Site was included in these same models as a predictor variable, to take account of general differences in PAAIB size, habitat, wider geographical features and any unknown factors. We did not include other predictors into the main model, because this would have reduced degrees of freedom on an already small number of replicates and it might have controlled away much of the variation that the study was designed to explain. To take these other factors into account and to reduce the risk of auto correlation or pseudo-replication due to the small sample size, covarying geographical characteristics (including altitude) and experimental design features (distance from boundary, camera field of vision (range in metres), number of survey days) were collected in order to test for homogeneity across treatments. These were then tested with a non-parametric Kruskal-Wallis-test to compare distribution and medians of each of these factors between treatments.

Trap rate data were used to analyse co-occupancy between humans and native species. Non-parametric Spearman's rank correlation tests in SPSS were used to explore these relationships. In order to reduce the risk of inflation of Type I errors through multiple testing, all possible associations were not tested; instead only correlations between humans and native species that were deemed relevant *a priori* were carried out. Bonferroni corrections were not used because only hypotheses of *a priori* interest were tested (correlation between humans and eight native species) and, with a limited amount of data, it was important not to inflate the Type II error rate through being over-conservative [43], [44].

## Results

### Detection rate

The 36 cameras in four PAs were operational for 9623 trap days. There were 1489 photographed events of target native species. Their breakdown is displayed in Tables 3 and 4. Fewer than 8% of these occurred at ORPI, while the remainder were distributed between BANWR (32.5%), NRD (22.4%) and CNM (36.1%). Artiodactyla only constituted just over half of all detections (51.7%) with Carnivora making up just under half (48.3%). Two thirds of these Carnivora detections were of Canidae and Felidae, while the remaining third were composed of smaller species families such as Procyonidae and Mustelidae. Bobcat were observed at more camera stations (83.3%) than any other species followed by coyote (80.6%) and deer species (either white-tailed deer or mule deer) (80.6%) with fox species (either kit fox or gray fox) observed at just under 75% of camera stations. 44.5% of all target native species detections were of a deer species. The next most frequently observed species were coyote (11.6%) and fox (10.7%). In spite of the high rate of deer detections,, It proved hard to tell the difference between the four skunk species so they were combined under their umbrella common name.

**Table 3:** Order, family and threat type trap rate summary by site and treatment at four protected areas in Arizona 2010–2011.

	Artiodactyla	Carnivora	Canidae/Felidae	Procyonidae/Mustelidae	Human	Invasive
ORPI	0.09	4.94	4.77	0.17	1.93	0.30
BA	9.12	10.53	7.74	2.79	1.89	0.75
NRD	8.72	5.95	4.49	1.45	5.81	4.14
CNM	13.66	8.06	4.51	3.55	2.34	0.24
NP	31.43	28.11	18.96	9.15	11.51	6.53
P	29.83	35.40	26.20	9.20	13.75	4.51
BE	33.06	24.91	19.47	5.45	11.25	5.71

*P = porous, NP = non-porous, BE = barrier-end. ORPI = Organ Pipe Cactus National Monument, BA = Buenos Aires National Wildlife Refuge, NRD = Nogales Ranger District (Coronado National Forest), CNM = Coronado National Memorial.*

**Table 4:** Species detected by distribution %, trap rate per 100 days, relative abundance and occupancy in four protected areas adjoining international boundaries 2010–2011.

Species	Bobcat	Puma	Coyote	Fox
**Total events**	139.0	46.0	173.0	159.0
**% events ORPI**	22.3	0.0	38.2	8.8
**% events BA**	35.3	21.7	39.3	44.0
**% events NRG**	19.4	73.9	12.1	12.6
**% events CNM**	23.0	4.3	10.4	34.6
**Trap rate per 100 days**	1.4	0.5	1.8	1.7
**Relative abundance**	9.3	3.1	11.6	10.7
**% Occupancy**	83.3	25.0	80.6	72.2

Species	Coati	Skunk	Deer	Collared Peccary
**Scientific name**	Nasua narica	Several species	Several species	Pecari tajacu
**Total events**	48.0	117.0	662.0	109.0
**% events ORPI**	0.0	0.9	0.3	0.0
**% events BA**	31.3	45.3	31.7	20.2
**% events NRG**	12.5	16.2	23.0	42.2
**% events CNM**	56.3	37.6	45.0	37.6
**Trap rate per 100 days**	0.5	1.2	6.9	1.1
**Relative abundance**	3.2	7.9	44.5	7.3
**% Occupancy**	27.8	61.1	80.6	61.1

*P = porous, NP = non-porous, BE = barrier-end. ORPI = Organ Pipe Cactus National Monument, BA = Buenos Aires National Wildlife Refuge, NRD = Nogales Ranger District (Coronado National Forest), CNM = Coronado National Memorial.*

There were 116 photographic events involving target invasive species; 72.2% of these occurred at NRD. Across all sites and all camera stations there was a mean detection rate of one target invasive every 82 days. 90% of the target invasive species detections were of cattle. Domestic dog (10%) constituted the remainder. Total target invasive species events were 8.4% of the total number of target native events, with a particularly high rate in NRD at which target invasive events were equivalent to 25.5% of target native events. Cattle and domestic dog had a trap rate of 1.08 pcd and 0.12 pcd respectively. Cattle appeared at 12 stations (33%) and domestic dogs at five stations (12.9%).

There were 283 photographic events involving humans, with 726 different individuals identified. 46.6% of these occurred at NRD with the remainder approximately equally distributed between the other sites. Across all sites and all camera stations there was a mean detection rate of one human event every 6.46 days. Total human events were equivalent to 19% of the total number of target native events, with a particularly high rate in ORPI and NRD at which human events were equivalent to 38.4% and 26.4% of target native events respectively. Undocumented Aliens (UDA) had a trap rate of 1.6 pcd. Combined human trap rate was 2.94 pcd. Humans appeared at 33 (91.6%) of camera stations, higher than bobcat, the leading native species at 83.3%. UDA appeared at 23 stations (62.8%). Mean group size for UDA was 4.6 individuals, with 5.8 for smugglers.The highest group size for a single event was 27 UDA at BANWR in May 2010.

### Design test

The Kruskal-Wallis tests showed no significant difference between the medians of survey days, boundary distance, camera field of vision and altitude between treatments across the selected PAAIB (Table 5). However the mean P to NP difference in elevation was 90 m and the mean P to BE difference was 68 m, but the overall elevation difference across the range of sites was 1285 m. So the treatment differences were equivalent to only 7% of the overall changes in elevation between sites. This difference arose by chance because of the small sample size and was influenced by the fact that most fencing along the international boundary stops in mountainous areas, as these are too difficult to build on. Although treatments were configured in a different east to west order between sites, the limited number of applicable PAAIB meant that the differences in altitude had to be accepted as they were, although they were somewhat mitigated by the inclusion of site as a predictor in the main model.

**Table 5:** Tests of homogeneity for camera trap stations between treatments. Non-parametric Kruskal-Wallis one-way analysis of variance.

Factor	p-value
Altitude	0.450
Camera view (m^2^)	0.0155
Distance to international boundary	0.582
Trap days	0.376

### Barrier treatment results

**Figure 2:**
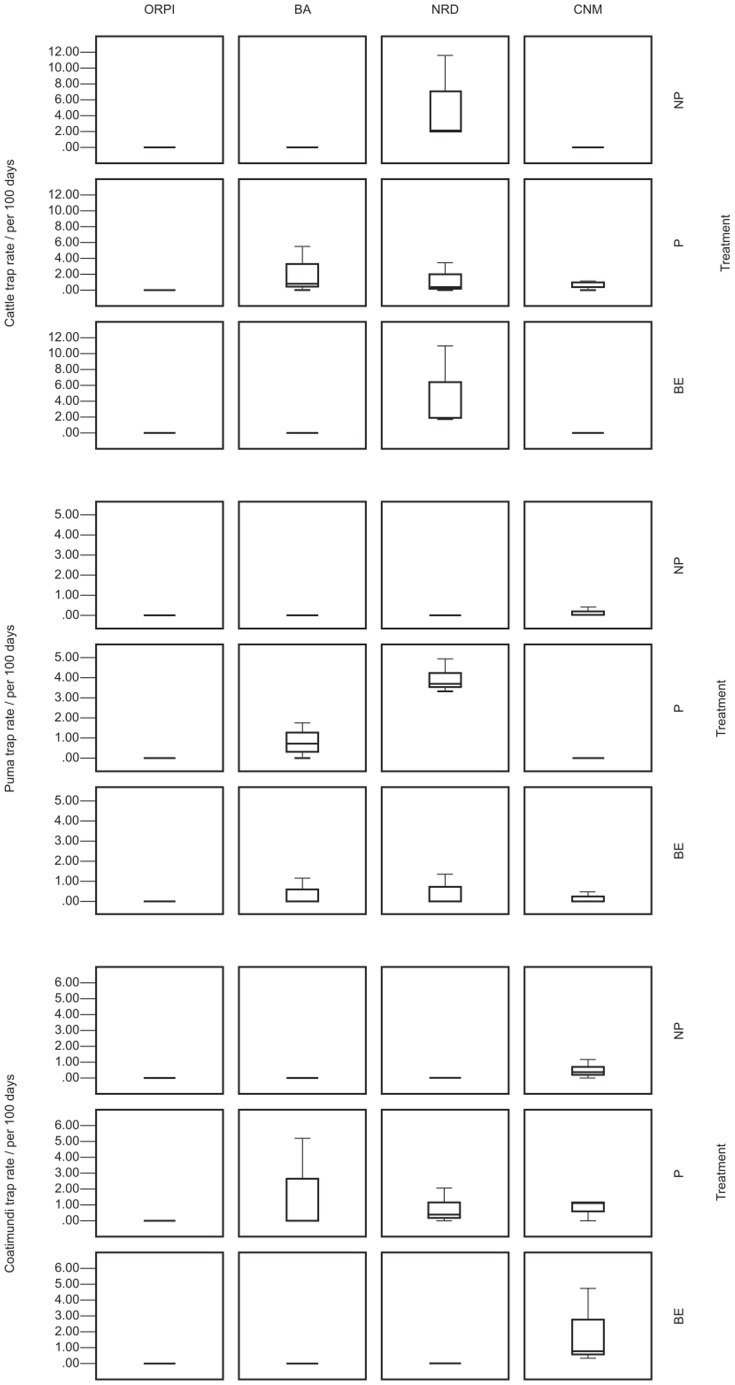
Mean trap rate for mammals in four PAs in Arizona, 2010–2011. Selected species include those which showed significant difference in treatment counts offset by the log of trap days) in porous (P), non-porous (NP) and barrier end (BE) treatments. Bars denote medians, boxes represent the interquartile ranges and whiskers show the full range.

Because of the low sample size and the elusive nature of many of the target species, there was a high chance of zero inflation and some convergence warnings appeared in the analyses. In order to ensure that the model was still suitable and therefore providing a meaningful test result, the model parameters for each species were checked to see if they reasonably matched the mean of the raw data. In particular we checked that the parameter value was not on the way to infinity or an infinitesimal fraction. In all cases this applied, suggesting a good model fit. There were significant results for both puma and coati *Nasua narica* in their relationship to treatment (they were detected more frequently in the P zones) and several more species in relation to site. (Table 6, Figure 2). Puma and coati were also analysed with distance from international boundary as a predictor - with no significant relationship.

**Table 6:** Trap rate difference test results between porous, non-porous and barrier-end treatments in four Arizona PAAIB 2010–2011.

Species	Deviance/df ratio	Treatment		Site	
		Wald Chi-Sq	Sig	Wald Chi-Sq	Sig
Bear	0.154	0.069	0.793	-	-
Bobcat	0.837	2.004	0.367	1.014	0.798
Puma	0.782	9.790	0.007*	6.412	0.041*
Coyote	1.004	1.154	0.562	10.524	0.015*
Fox	1.815	0.535	0.765	8.428	0.038*
Coati	1.111	9.685	0.008*	7.544	0.023*
Skunk	0.931	3.456	0.178	13.406	0.004*
Deer	0.699	0.136	0.934	33.139	<0.001*
Collared peccary	0.899	1.685	0.431	2.282	0.319
Cattle	1.221	6.303	0.043*	22.376	<0.001*
Dog	0.727	0.650	0.420	2.389	0.122
Horse	0.453	-	-	-	-
UDA	1.426	3.481	0.175	14.511	0.002*
Smuggler	0.811	4.189	0.123	0.396	0.820
Other Human	0.797	1.892	0.388	6.656	0.084

*Tests were carried out using a negative binomial GLM. Deviance and degrees of freedom ratio illustrate goodness of model fit and the Wald statistic was used to test the significance of model terms. P = porous, NP = non-porous, BE = barrier-end. ORPI = Organ Pipe Cactus National Monument, BA = Buenos Aires National Wildlife Refuge, NRD = Nogales Ranger District (Coronado National Forest), CNM = Coronado National Memorial. Hyphens show where the data could not be computed “due to numerical problems” likely due to the low number of stations and sites registering detections.*

*Denotes significant results at p values equal to or less than 0.05.

### Species correlations

There was a positive correlation between UDA and puma activity (rho = 0.388, n = 36, p = 0.019). Combined human categories correlated positively with puma (rho = 0.505, n = 36, p = 0.002) and collared peccary (rho = 0.358, n = 36, p = 0.032). It is notable that none of the correlation coefficients were above 0.505.

## Discussion

### Effects on native species detection

This research was directed at understanding whether “thickening” infrastructure on an international boundary perimeter of a PA is likely to inhibit movement and restrict access to resources for certain native species. We also wanted to investigate whether boundary barriers can prevent incursion from humans and invasive species.

Puma and coati detection in dry river beds were significantly different between treatments, with both of them displaying higher trap rates in the P zones. Both of the impacted species were Carnivora, with no significant differences for any native Artiodactyla. It is hard to tell if these changes illustrate a rise in population in the P zone, a collapse in population in the NP zone or a migration of population from NP to P or a combination of these. The answer may well be different for different species. For example, those with a wide dispersal ability, such as puma, may move their home range to another area in response to disturbance, while smaller species with limited dispersal ability, such as coati, may not be able to move home range and may therefore be forced to move within a restricted, overlapping area, leading to diminished resources and a possible collapse in population. These two species have different morphology, spatial and habitat requirements. This implies that the barrier and its direct and indirect effects can have a range of influences on a range of species across a range of trophic scales.

This supports literature which shows that barriers dissect, filter, eliminate or complicate movement [45] and can influence small mammals [46] and large mammals alike. This can leave them vulnerable to isolation, stochastic events and extinction. Furthermore, Strongly Interacting Species (SIS), such as carnivores, feature relatively highly amongst endangered species [47], so they may be particularly vulnerable to these threats or any stochastic fluctuations that may follow. So these impacts on puma and coati may have serious implications for their behaviour and populations as well as those with whom they interact.

Six of the eight most detected species did not have a significantly different detection rate between treatments. It was expected that not all target species would show a significant difference between treatments because of their different spatial and resource requirements and the different ways in which the barrier might affect these. It is likely that these were in part due to their generalist nature and ability to adapt behaviour to the changed circumstances. For example deer [48], bobcat [49], coyote [50] and fox are considered to be generalist species. It is possible that without competition from puma, these species may fill any ecological niche left by them in certain areas. It may also be possible that certain species, such as skunk, had a limited home range that was not directly affected by the barrier.

Although not apparently impacted directly, these six species may be indirectly impacted in the longer term. Because Carnivora may influence other species through predation, any alteration in the behaviour of one species can have wide-scale knock-on effects for ecosystem [51], [52]. Even small Carnivora can influence other Orders [53], [54], such as Artiodactyla, with an effect on plant composition [55] or plant seedlings, which may in turn affect breeding songbirds [56]. Because of these complex interactions, when one of these groups rises or falls in abundance there can be profound effects for others [57]. This matters because any changes may disturb the delicate balance and interaction of life forms within an ecosystem. As a result large and widely distributed carnivore populations are important for the maintenance of biodiversity [57] and their rarity or absence can lead to changes or simplifications in ecosystem structure. These might include “structural or compositional modifications, alterations in the import or export of nutrients, loss of resilience to disturbance, and decreases in native species diversity” [47].

### Effects on transboundary anthropogenic detection

Over four PAs, human activity did not appear to be affected by treatment type. These results are supported by other research, which shows that international boundary security infrastructure has little or no effect on the attitude of UDA to international boundary crossings [58]. Therefore impacts wrought by humans are likely to be similar in both P and NP areas. The non-significant impact of treatment on human detections is most likely evident because of the factors that drive US-Mexico migration, which are strong enough to overcome static prevention measures.

### Effects on transboundary invasive species detection

Domestic dogs were not detected differently between treatments and the overall detection rate was low. This infers that the barrier plays little part in restricting their activity, probably because it is extremely difficult to wholly exclude invasive species from a PAAIB, if they reside in its vicinity. It was widely acknowledged by PAAIB staff that the majority of the domestic dogs present in the surveyed PAAIB originated from the Mexican side of the international boundary. For example one dog was photographed as part of a pack inside ORPI but was also directly photographed inside Mexico. Cattle were detected significantly more in the P treatment areas while NP has the highest overall count. Therefore the significant effect of treatment for cattle appears to be at odds with the plotted data (Figure 2), where this pattern only holds for NRD (site 3), with BA (sites 2) and CNM (site 4) showing a different trend (P highest) and ORPI (site 1) having zero counts for all treatments. Indeed, although the mean count for NP is highest overall, the overall treatment effect is not robust: the site:treatment interaction has a significant effect (chi-squared = 14.34, d.f. = 6, p = 0.026) and, given this would reduce the sample size to three for each treatment, it was not worthwhile breaking the analysis down to separate tests for each site. It is therefore only possible to conclude that the effect of treatment on cattle is variable and site specific. In any case the majority of the cattle detected were presumed to originate from the US side of the international boundary, because NRD allows cattle grazing. It is likely that the barrier kept them within the US, and even semi-porous fencing (such as vehicle barriers or barbed wire) would prohibit transboundary movement of these species. As a result they cannot be deemed to be transboundary invasive species.

### Native species and human correlation

Correlation between humans and both puma and peccary in dry river beds are important because they show that native species may be influenced by human activity. This may well be related to the fact that both humans and puma are more active in the P zones. However, whatever the cause, it has been estimated that a typical illegal migrant or undocumented alien (UDA) leaves 4 kg of solid waste each day [59] and causes some cactus damage [60]. Others estimate that every 1000 unauthorized immigrants create 72 m of new trail, 656 m^2^ of disturbed habitat, 50 kg of litter, 11 campfires and 1.7 ha of wildfire damage [61]. These anthropogenic impacts may exacerbate any changes to ecosystem functioning caused by the barrier effects themselves. Low rho values between certain species may be explained by the zero inflation in the sample, caused by the elusive nature of many of the target species. Non-significant results may also be influenced by this issue.

### Wider implications of research

These results indicate that intermittently closed boundaries do not deliver protection from transboundary anthropogenic impacts but that they also limit resources for certain native species. To this extent the status quo delivers the worst of both worlds for biodiversity. Based on earlier studies, we did expect some disruption for some species, although we did not know which ones, but we did expect barriers to exclude humans to some significant degree.

At the other end of the “thickening” scale lie transboundary conservation (TBC) schemes. TBC initiatives seek to cooperatively protect and maintain ecosystems and/or species that straddle international boundaries. A pair of internationally adjoining PAAIB may decide to process, identify and map a shared ecosystem [62] and then adopt and adhere to an agreed co-management strategy. Equally there may be more informal, local arrangements between PAAIB staff. In each case, TBC proponents highlight the potential for spatial, management, socio-economic and political benefits through transboundary cooperation. Such schemes can be very effective, but it is clear that the correct choice of strategy for PAAIBs is both species and context dependent [19] which may well explain the differences in opinion between the proponents of closed and open conservation schemes [10], [15].

Management options are not a simple choice between open or closed and invasion or isolation, because it is practically impossible to design a fully closed system. There may always be terrestrial, sub-terrestrial, waterborne and airborne modes of movement by which some human activities and some invasive species may enter an ecosystem. Likewise a fully open system (even in a TBPA project) is unlikely to exist, because there may always be impediments to movement, whether anthropogenic or not, for certain species. The management choices are more about selecting a position on the open-closed continuum that provides maximum access to resources and maximum protection for the species (or habitat) of primary conservation importance while taking into account socio-economic and geographical factors.

## References

[1] Naughton-Treves L, Holland MB, Brandon K (2005) The role of protected areas in conserving biodiversity and sustaining local livelihoods. Annu Rev Environ Resour 30: 219–252.

[2] Struhsaker TT, Struhsaker PJ, Siex KS (2005) Conserving Africa's rain forests: problems in protected areas and possible solutions. Biol Conserv 123: 45–54.

[3] Andam KS, Ferraro PJ, Pfaff A, Sanchez-Azofeifa GA, Robalino JA (2008) Measuring the effectiveness of protected area networks in reducing deforestation. Proc Natl Acad Sci USA 105: 16089–16094. 18854414 10.1073/pnas.0800437105 PMC2567237

[4] Maiorano L, Falcucci A, Boitani L (2008) Size-dependent resistance of protected areas to land-use change (2008). Proc R Soc Lond 275: 1297–1304. 10.1098/rspb.2007.1756 PMC2602674 18319213

[5] Gaston KJ, Jackson SE, Cantu-Salazar L, Cruz-Pinon G (2008) The ecological performance of protected areas. Annu Rev Ecol, Evol Syst 39: 93–112.

[6] Craigie ID, Baillie JEM, Balmford A, Carbone C, Collen B, Green RE, Hutton JM (2010) Large mammal population declines in Africa's protected areas. Biol Conserv 143: 2221–2228.

[7] Lysenko I, Besancon C, Savy C (2007) Global list of transboundary protected areas. UNEP-WCMC, Cambridge, UK.

[8] Van Schoik DR, Lelea E, Cunningham J (2007) Transboundary biodiversity: turning a potential tragedy into a true partnership. In: Hoffman K (ed) US-Mexican border environment and transboundary ecosystem management, Monograph 15, Chapter 1. The Southwest Consortium for Environ. Research and Policy (SCERP), Phoenix, USA.

[9] Brockington D (2002) Fortress Conservation: The Preservation of the Mkomazi Game Reserve, Tanzania.

[10] Packer C, Loveridge A, Canney S, Caro T, Garnett ST, et al (2013) Conserving large carnivores: dollars and fences. Ecol Lett 16: 635–641. 23461543 10.1111/ele.12091

[11] Andreas P (2003) Redrawing the line – borders and security in the twenty-first century. Int Sec 28: 78–111.

[12] Newman D (2006) Borders and bordering – towards an interdisciplinary dialogue. Eur J Soc Theor 9: 171–186.

[13] Lasky JR, Jetz W, Keitt TH (2011) Conservation biogeography of the US-Mexico border: a transcontinental risk assessment of barriers to animal dispersal. Divers Distrib 17: 673–687.

[14] De Fries R, Hansen A, Newton AC, Hansen MC (2005) Increasing isolation of protected areas in tropical forests over the past twenty years. Ecol Appl 15: 19–26.

[15] Creel S, Becker MS, Durant SM, M'Soka J, Matandiko W, et al (2014) Conserving large populations of lions - the argument for fences has holes. Ecol Lett doi:101111/ele12145. (In Press) 10.1111/ele.12145 23837659

[16] McShane TO, Hirsch PD, Trung TC, Songorwa AN, Kinzig A, et al (2011) Hard choices: making trade-offs between biodiversity conservation and human well-being. Biol Conserv 144: 966–972.

[17] Kapos V, Balmford A, Aveling R, Bubb P, Carey P, et al (2009) Outcomes, not implementation, predict conservation success. Oryx 43: 336–342.

[18] Busch J (2008) Gains from configuration: the transboundary protected area as a conservation tool. Ecol Econ 67: 394–404.

[19] Ferguson K, Hanks J, eds. (2010) Fencing Impacts: A review of the environmental, social and economic impacts of game and veterinary fencing in Africa with particular reference to the Great Limpopo and Kavango-Zambezi Transfrontier Conservation Areas. Pretoria: Mammal Research Institute. Available: http://www.wcs-ahead.org/gltfca_grants/grants.html

[20] Novosseloff A, Neisse F, Rufin JC (2007) Des Murs Entre Les Hommes. La Documentation Francaise. France.

[21] Senate Bill 744 The Border Security, Economic Opportunity and Immigration Modernisation Act. United States Senate S 744.

[22] Ewers RM, Didham RK, Fahrig L, Ferraz G, Hector A, et al (2000) A large-scale forest fragmentation experiment: the Stability of Altered Forest Ecosystems project. Proc R Soc Lond 366: 3292–3302. 10.1098/rstb.2011.0049 PMC3179633 22006969

[23] Oksanen L (2001) Logic of experiments in ecology: is pseudoreplication a pseudoissue? Oikos 94: 27–38.

[24] McGarigal K, Cushman SA (2002) Comparative evaluation of experimental approaches to the study or habitat fragmentation effects. Ecol Appl 12: 335–345.

[25] Myers N, Mittermeier RA, Mittermeier CG, da Fonseca GAB, Kent J (2000) Biodiversity hotspots for conservation priorities. Nature (London) 403: 853–858. 10706275 10.1038/35002501

[26] Omernik JM (1987) Ecoregions of the conterminous United States. Map (scale 1∶7,500,000). Ann Assoc Am Geogr 77: 118–125.

[27] Bailey R (1989/1993) Ecoregions. USGS Public Warehouse PP 1650-E. Available: http://pubs.usgs.gov/pp/p1650-e/bailey_maps.html.

[28] Murcia C (1995) Edge effects in fragmented forests – implications for conservation. Trends Ecol Evol 10: 58–62. 21236953 10.1016/S0169-5347(00)88977-6

[29] Debinski DM, Holt RD (2000) A survey and overview of habitat fragmentation experiments. Conserv Biol 14: 342–355.

[30] Chase JM, Abrams PA, Grover JP, Diehl S, Chesson P, et al (2002) The interaction between predation and competition: a review and synthesis. Ecol Lett 5: 302.

[31] Bronstein JL (1994) Our current understanding of mutualism. Q Rev Biol 69: 31–51.

[32] Wonham MJ, Lewis MA, Renclawowicz J, Van den Driessche P (2006) Transmission assumptions generate conflicting predictions in host-vector disease models: a case study in West Nile virus. Ecol Lett 9: 706–725. 16706915 10.1111/j.1461-0248.2006.00912.x

[33] Bruno JF, Stachowicz JJ, Bertness MD (2003) Inclusion of facilitation into ecological theory. Trends Ecol Evol 18: 119–125.

[34] Hooper DU, Chapin FS, Ewel JJ, Hector A, Inchausti P, et al (2005) Effects of biodiversity on ecosystem functioning: a consensus of current knowledge. Ecol Monogr 75: 3–35.

[35] McDonald W, St. Clair CC (2004) Elements that promote highway crossing structure use by small mammals in Banff National Park. J Appl Ecol 41: 82–93.

[36] Wolff JO, Schauber EM, Edge WD (1997) Effects of habitat loss and fragmentation on the behaviour and demography of gray-tailed voles. Conserv Biol 11: 945–956.

[37] Smallwood KS (1999) Scale domains of abundance amongst species of mammalian Carnivora. Environ Conserv 26: 102–111.

[38] Karanth KU (1995) Estimating tiger Panthera tigris populations from camera-trap data using capture–recapture models. Biol Conserv 71: 333–338.

[39] Hilty JA, Merenlender AM (2004) Predators in riparian corridors and vineyards. Conserv Biol 18: 126–135.

[40] Harris G, Thompson R, Childs JL, Sanderson JG (2010) Automatic storage and analysis of camera trap data. Bull Ecol Soc Am 91: 352–360.

[41] Venables WN, Ripley BD (2002) Modern Applied Statistics with S (Statistics and Computing). Fourth Edition. Springer, New York, USA.

[42] R Core Team (2013). R: A language and environment for statistical computing. R Foundation for Statistical Computing. Vienna, Austria. URL http://www.R-project.org/

[43] Nakagawa S (2004) A farewell to Bonferroni: the problems of low statistical power and publication bias. Behav Ecol 15: 1044–1045.

[44] Ruxton GD, Beauchamp G (2008) Time for some a priori thinking about post hoc testing. Behav Ecol 9: 690–693.

[45] McDonald WR, St Clair CC (2004) The effects of artificial and natural barriers on the movement of small mammals in Banff National Park Canada. Oikos 105: 397–407.

[46] Valenzuela D, Macdonald DW (2002) Home range use by white-nosed coatis Nasua narica: limited water and a test of the resource dispersion hypothesis. J Zool 258: 247–256.

[47] Soulé ME, Estes JA, Berger J, Del Rio CM (2003) Ecological effectiveness: conservation goals for interactive species. Conserv Biol 17: 1238–1250.

[48] Abbas F, Morellet N, Hewison AJM, Merlet J, Cargnelutti B, et al (2011) Landscape fragmentation generates spatial variation of diet composition and quality in a generalist herbivore. Oecologia 167: 401–411. 21519885 10.1007/s00442-011-1994-0

[49] Peers MJL, Thornton DH, Murray DL (2012) Reconsidering the Specialist-Generalist Paradigm in Niche Breadth Dynamics: Resource Gradient Selection by Canada Lynx and Bobcat. PLoS ONE 7 (12) e51488 doi:10.1371/journal.pone.0051488 23236508 10.1371/journal.pone.0051488 PMC3517500

[50] Bozarth CA, Hailer F, Rockwood LL, Edwards CW, Maldonado JE (2011) Coyote colonization of northern Virginia and admixture with Great Lakes wolves. J Mamm 92: 1070–1080.

[51] Karki SM, Gese EM, Klavetter ML (2007) Effects of coyote population reduction on swift fox demographics in southeastern Colorado. J Wildl Manage 71: 2707–2718.

[52] Dalerum F, Somers MJ, Kunkel KE, Cameron EZ (2008) The potential for large carnivores to act as biodiversity surrogates in southern Africa. Biodivers Conserv 17: 2939–2949.

[53] Korpimaki E, Norrdahl K (1998) Experimental reduction of predators reverses the crash phase of small-rodent cycles. Ecology 79: 2448–2455.

[54] Wang H, Fuller TK (2003) Food habits of four sympatric carnivores in southeastern China. Mammalia 67: 513–519.

[55] Waller DM, Alverson WS (1997) The white-tailed deer: a keystone herbivore. Wildl Soc Bull 25: 217–226.

[56] Cote SD, Rooney TP, Tremblay JP, Dussault C, Waller DM (2004) Ecological impacts of deer overabundance. Annu Rev Ecol Evol Syst 35: 113–147.

[57] Soulé ME, Estes JA, Miller B, Honnold DL (2005) Strongly interacting species: conservation policy management and ethics. BioScience 55: 168–176.

[58] Cornelius W, Salehyan I (2007) Does border enforcement deter unauthorised immigration? The case of Mexican migration to the United States. Regu Gov 1: 139–153.

[59] Moya H, Cordova A (Ed), de la Parra CA (Ed) (2007) Possible impacts of border fence construction and operation on fauna. A Barrier to our Shared Environment: the Border Fence Between the United States and Mexico. National Institute of Ecology, Mexico City, Mexico.

[60] Sundberg J, Kaserman B (2007) Cactus carvings and desert defecations: embodying representations of border crossings in protected areas on the Mexico-US border. Environ Plan Soc Space 25: 727–744.

[61] McIntyre DL, Weeks JR (2002) Environmental impacts of illegal immigration on the Cleveland national forest in California. Prof Geogr 54: 392–405.

[62] Hamilton LS (1996) Transborder protected area co-operation. In: Cerovsky J (ed) Biodiversity Conservation in Transboundary Protected Areas in Europe. Ecopoint, Prague, Czech Republic.

